# X-linked hypophosphatemic rickets: an Italian experts’ opinion survey

**DOI:** 10.1186/s13052-019-0654-6

**Published:** 2019-05-31

**Authors:** F. Emma, M. Cappa, F. Antoniazzi, M. L. Bianchi, I. Chiodini, C. Eller Vainicher, N. Di Iorgi, M. Maghnie, A. Cassio, A. Balsamo, F. Baronio, L. de Sanctis, D. Tessaris, G. I. Baroncelli, S. Mora, M. L. Brandi, G. Weber, A. D’Ausilio, E. P. Lanati

**Affiliations:** 1 Division of Nephrology, Department of Pediatric Subspecialties, Children’s Hospital Bambino Gesù, IRCCS, Piazza Sant’Onofrio 4, 00165 Rome, Italy; 2 Endocrinology Unit, Bambino Gesù Children’s Hospital, Rome, Italy; 3 Department of Surgery, Dentistry, Pediatrics and Gynecology, Pediatric Division, University of Verona, Borgo Roma Hospital, Verona, Italy; 4 Experimental Laboratory for Children’s Bone Metabolism Research, Bone Metabolism Unit, Istituto Auxologico Italiano IRCCS, Milan, Italy; 5 Unit for Bone Metabolism Diseases and Diabetes & Lab of Endocrine and Metabolic Research, Istituto Auxologico Italiano, IRCCS, Milan, Italy; 6 Dept. of Clinical Sciences & Community Health, University of Milan, Milan, Italy; 7 Unit of Endocrinology, IRCCS Cà Granda Foundation, Maggiore Policlinico Hospital, Milan, Italy; 8 Department of Neuroscience, Rehabilitation, Ophthalmology, Genetics, Maternal and Child Health Department of General and Specialist Pediatric Sciences, Pediatric Clinic, IRCCS Giannina Gaslini Institute, University of Genova, Genova, Italy; 9 Department of Medical & Surgical Sciences, Pediatric Unit, S. Orsola Malpighi University Hospital, Bologna, Italy; 10 Department of Public Health and Pediatric Sciences, University of Torino - Regina Margherita Children Hospital, Torino, Italy; 11 Pediatric Unit, Department of Obstetrics, Gynecology and Pediatrics, University Hospital of Pisa, Pisa, Italy; 12 Department of Pediatrics, IRCCS San Raffaele Hospital, Vita-Salute San Raffaele University, Milan, Italy; 13 Metabolic Bone Diseases Unit, Department of Surgery and Translational Medicine, Careggi University Hospital, University of Florence, Florence, Italy; 14 MA Provider, Milan, Italy

**Keywords:** FGF23, Osteomalacia, Vitamin D, Phosphate, Rickets

## Abstract

**Background:**

X-linked hypophosphatemic rickets (XLH) is the first cause of inherited hypophosphatemia and is caused by mutation in the *PHEX* gene, resulting in excessive expression of the phosphaturic factor FGF23. Symptoms are mainly related to rickets in children and osteomalacia in adults and cause several complications that can be highly invalidating. Due to its rarity, XLH is poorly known and diagnosis is frequently delayed. Conventional treatment is based on oral phosphate salts supplementation and activated vitamin D analogs, which however, cannot cure the disease in most cases.

**Objective:**

Due to the low prevalence of XLH, an experts’ opinion survey was conducted across Italian centers to collect data on XLH and on its management.

**Methods:**

A questionnaire was developed by a group of experts to collect data on XLH epidemiology, diagnosis and treatment in Italy.

**Results:**

Data from 10 Italian centers (nine of which pediatric) on 175 patients, followed between 1998 and 2017, were included in the survey. Most patients were followed since childhood and 63 children became adults during the investigated period. The diagnosis was made before the age of 1 and between 1 and 5 years in 11 and 50% of cases, respectively. Clinically apparent bone deformities were present in 95% of patients. These were ranked moderate/severe in 75% of subjects and caused growth stunting in 67% of patients. Other frequent complications included bone pain (40%), dental abscesses (33%), and dental malpositions (53%). Treatment protocols varied substantially among centers. Nephrocalcinosis was observed in 34% of patients. Tertiary hyperparathyroidism developed in 6% of patients.

**Conclusions:**

XLH remains a severe condition with significant morbidities.

## Background

X-linked hypophosphatemic rickets (XLH) (OMIM 307800) is a rare disease caused by mutations in the *PHEX* gene. The gene encodes for an endopeptidase that is primarily expressed on the surface of osteoblast, osteocytes, odontoblasts and cementoblasts [1, 2]. The disease follows an X-linked mode of transmission with dominant expression, and represents the first cause of hypophosphatemic rickets [1]. Female carriers usually present symptoms similar to male patients [3]. Unless patients are identified by family screening, they are usually diagnosed in the first years of life after noticing bowing of the lower legs, delayed walking, abnormal walking gait, and/or growth stunting. The disease may be initially misdiagnosed for vitamin D deficiency, but biochemical investigations and in some cases, lack of response to vitamin D supplements usually allow the diagnosis. Typically, patients show at diagnosis low serum phosphate levels secondary to increased phosphaturia, elevated alkaline phosphatase, normal parathyroid hormone (PTH) levels and low-normal urine calcium. X-rays demonstrate typical features of rickets without significant bone resorption, as opposed to rickets secondary to vitamin D deficiency [1, 4]. Growth plates of long bones have a cupped appearance, with increased thickness and irregularities. Positive family history often facilitates the diagnosis, but de novo mutations occur frequently and some adults lack overt features of XLH [5].

Other frequent symptoms in children include rachitic rosary, dolichocephaly with parietal flattening and frontal bossing, and bone pain. More rarely, patients are diagnosed with craniosynostosis, type 1 Chiari malformations, intracranial hypertension or hearing impairment [5, 6]. The majority of children with overt XLH grow asymmetrically. They have slightly decreased sitting height and markedly decreased leg length. Patients often have dental symptoms secondary to dental malpositions and/or to hypomineralization of dentine and hypoplasia of the enamel, which cause frequent abscesses [1, 5, 6].

Adult patients usually present with symptoms secondary to defective bone mineralization (osteomalacia) and often complain of bone and joint pain [7]. They frequently develop pseudofractures, as well as invalidating osteoarthropathies that involve predominantly the ankles, knees and sacroiliac joints [7]. They can also develop enthesopathy, which is caused by calcification of tendons and ligaments. These are particularly prominent in the ankles, knees, pelvic joints and spine [8, 9].

The above symptoms highlight the severity of the disease, which may not be recognized due to its rarity. For this same reason clinical and biochemical data on large cohorts of patients are scanty.

On these premises, we have conducted a survey in ten Italian reference centers for the diagnosis and treatment of XLH, to evaluate the epidemiology of the disease, diagnostic challenges, treatment attitudes, organization of care and disease burden.

## Methods

A questionnaire was developed by a group of experts to collect retrospective data on XLH in Italy. The experts met initially in September 2016 and subsequently twice to elaborate the questionnaire, analyze the initial results and solve open issues to finalize data collection. The survey was limited to data that could be collected reliably from medical charts. Single patient data were not transferred; only aggregate data from each center were collected, to protect privacy. Patients were included in the study if the following data were retrievable from clinical charts: diagnosis, epidemiology and treatment pattern. Patients were excluded if such data were not available.

The diagnosis of XLH was based on genetic confirmation of a PHEX mutation, or, if not available, was based on symptoms, family history, radiological evidence of rickets, and laboratory tests.

Ten units were identified in North and Central Italy after screening pediatric, endocrinology and nephrology units for their expertise in XLH, as well as regional registries for rare diseases.^1^ The centers included in the study were selected based on: (i) number of patients treated every year and inclusion in the National Network for rare diseases. The questionnaire investigated the following disease areas: epidemiology, diagnosis, disease severity, treatment, organization of care.

Quality of life data were not available in the charts, therefore, the impact of the disease burden was estimated by the clinicians, using a 5-point scale (1: low impact, 5: high impact), inspired from the EUROQOL questionnaire, exploring various dimensions of daily life (functional capacity, mobility, usual activities, pain/discomfort).

Descriptive statistical analyses were used to illustrate data. Results are expressed as mean ± standard deviation (SD), median and range or percentage, as appropriate.

## Results

Data from 175 patients that were followed between 1998 and 2017 in the 10 participating centers were included in the survey (Table 1).

**Table 1:** Total number of patients managed and origin

	Lombardia (Milan - 3 centres)	Piemonte (Torino)	Liguria (Genoa)	Emilia-Romagna (Bologna)	Toscana (2 centres: Pisa and Florence)	Lazio (Rome - 2 centres)	TOT
Total number of patients	44	19	3	18	46	45	175
Patients from the same region	33	14	0	4	20	30	101
*%*	*75%*	*74%*	*0%*	*22%*	*43%*	*67%*	*58%*
Patients from other regions	11	5	3	14	26	15	74
*%*	*25%*	*26%*	*100%*	*78%*	*57%*	*33%*	*42%*

Most centers were pediatric units, therefore data on adult patients were limited. Twenty-two patients were diagnosed before 1998 and followed afterwards. Of the 153 patients (75% males and 25% females) diagnosed after 1998, 135 were pediatric (0–17 years of age). For most criteria, data were available for all patients; however, some data could not be traced back from all medical charts or were restricted to pediatric patients.

### Diagnosis

The distribution of the age at diagnosis is reported in Table 2. Such info was available for 150 patients only; of those, over 60% were diagnosed between 1 and 5 years No trends towards earlier diagnosis in recent years was observed by comparing patients diagnosed after and before 2012 (data not shown).

**Table 2:** Age at diagnosis in patients *diagnosed after 1998*

Age at diagnosis	Number of patients (1998–2017)	%
*0–1 years*	*17*	*11%*
*1–5 years*	*75*	*50%*
*5–12 years*	*35*	*23%*
*12–17 years*	*6*	*4%*
*≥18 years*	*17*	*11%*
*TOTAL*	*150*	*100%*

A genetic test was performed in 65 patients and a pathogenic *PHEX* mutation was confirmed in 56 cases (86%). For the reminder of the cohort, the diagnosis was based on clinical grounds. Physicians from the 10 centers were hence asked to rank the most important features leading to the diagnosis of XLH. These included rickets, hypophosphatemia and, when available, a positive family history. Interestingly, 17% of patients had been diagnosed with vitamin D deficiency prior to the diagnosis of XLH. Overall, positive family history was present in 33% (54/164) of patients in which the information was available.

### Symptoms

**Fig. 1:**
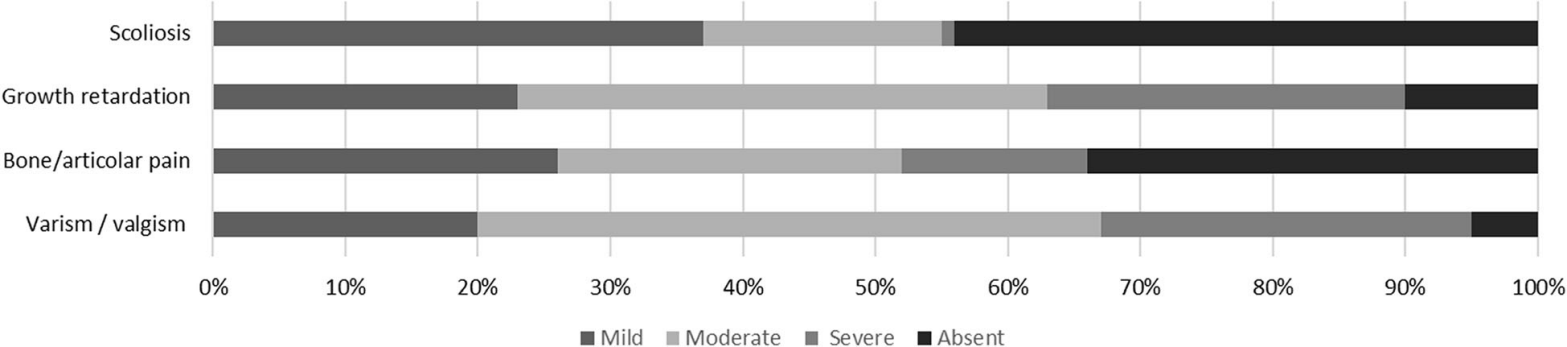
Severity of symtoms

Most (94,8%) patients had lower leg deformities, which were ranked severe in 28%, moderate in 47%, and mild or absent in the remainder 25% of patients. “Severe” deformities referred to overt *genu valgus* or *genu varum* that impaired normal walking, “moderate” to less important deformities with mild or no walking impairment, and “mild” to minor limb deformities with regular joint alignment (Fig. 1).

Bone pain was reported in two third (65,6%) of patients but was ranked as mild to moderate in most (79,3%) cases. More than half (67%) of patients had growth retardation (height lower than − 2 SD). Scoliosis or osteopenia was seldom reported. Pathological fractures were observed in nearly one third of patients. Craniosynostosis was reported in 5% of the cohort. Dental malpositions were present in half of patients (53%) and approximately one third (33%) had experienced at least one dental abscess. Hearing impairment and neurological problems were infrequent (observed in 5 and 9% of cases, respectively - Table 3).

**Table 3:** Prevalence of other XLH-related symptoms

Disease	Yes	No
Reduced Muscular Force	*66%*	*34%*
Dental malpositions	*53%*	*47%*
Dental abscess	*33%*	*67%*
Osteopenia	*16%*	*84%*
Pathological fractures	*13%*	*87%*
Neurological problems	*12%*	*88%*
Hearing impairment	*7%*	*93%*
Craniosynostosis	*6%*	*94%*

### Medical and orthopedic treatment

All centers prescribed conventional treatment to all pediatric patients, including oral phosphate supplements and active vitamin D analogues, namely calcitriol and alfacalcidol. The latter was generally preferred in young children because liquid solutions are commercially available in Italy. In some centers non-active vitamin D supplements were also prescribed. Doses were modified according to age, but the dose range was considerably wide. For example, the dose of calcitriol for school age children ranged from 0.25 to 2.0 mcg/day. In some centers, doses of vitamin D was calculated according to weight, while other centers used fixed doses and adapted treatment according to the biochemical response. Phosphate salt prescription was equally very variable. For school age children, the dose varied from 15 to 60 mg/Kg/day. Phosphate was prescribed in different pharmacologic preparations, including using IV solution for oral administration, galenic solutions (Joulie or modified Joulie solutions), and commercially available tablets or liquid solutions, some of which were imported from other European countries. The frequency of uptake also varied, but most centers prescribed four doses per day in preadolescent patients and usually reduced the frequency to 2–3 doses per day in adolescents. Two centers did not recommend systematic treatment in adults, while the remaining centers advocated to continue low dose treatment with phosphate supplements and/or vitamin D, irrespectively to the presence of symptoms.

Likewise, metabolic targets for optimal disease control were, to some extent, different between centers. Unanimously, normalization of alkaline phosphatase was the main biochemical marker of a satisfactory treatment, with optimal target values within the normal range and acceptable values below 1.5-fold the upper limit of normal range for age. All experts tried to keep serum calcium and PTH levels within the normal range. In six centers, serum phosphate levels did not represent a target for treatment. Nonetheless, four centers aimed at serum phosphate levels above a given threshold, ranging 2–3 mg/dl.

Complications of conventional treatment include nephrocalcinosis and hyperparathyroidism. Retrospective evaluation of renal ultrasound did not allow ranking the severity of nephrocalcinosis. Likewise, hyperparathyroidism was difficult to assess and for consistency, only patients requiring surgery or treatment with cinacalcet for tertiary hyperparathyroidism were recorded. Overall, nephrocalcinosis was reported in 34% of patients, while tertiary hyperparathyroidism was developed in 6%.

From the orthopedic standpoint, half of patients had undergone at least one surgical procedure. These included osteotomies and epiphysiodesis, with the latter procedure being more frequently used in recent years. More detailed analyses were not possible due to the multicentric nature of the survey and changes in the clinical practice and indications for surgery over the years, including the type of procedure.

### Professional caregivers

Most pediatric patients were followed by pediatric endocrinologists (7/10), pediatricians with a special interest in XLH (2/10) and pediatric nephrologists (1/10). In all cases a multidisciplinary team, including orthopedic surgeons, radiologists, dentists, and possibly geneticists were activated. Neurologists, neurosurgeons, physical therapists were also involved in the team, though less frequently.

During the time frame of the survey, 63 patients had reached adult age. Of these, 31 continued to be followed in pediatric centers (11 aged 18–25 years, and 22 aged > 25 years). The remaining adult patients were addressed to adult endocrinologists, nephrologists or rheumatologists.

### Disease burden

Quality of life (QoL) data were not available due to the retrospective nature of the study. Nonetheless, experts participating in the study were asked to estimate the impact of the disease on patients, using a 5-point scale (1: low impact, 5: high impact), inspired from the EUROQOL questionnaire, which explored several dimensions of daily life (functional capacity, mobility, usual activities, pain/discomfort). The estimated impact of the disease on daily life ranked on average 4.5 for patients with severe disease, 3.7 for patients with moderate disease and 1.8 for those with mild symptoms. “Severe” disease corresponded to patients with severe leg deformities as defined above and/or major complications, “moderate” to moderate bone changes and limited complications, such as dental malpositions, and “mild” to the remaining patients. As expected, rickets secondary to vitamin D deficiency ranked best (1.8). Osteopetrosis ranked worst (4.61) and XLH had and intermediate score of 3.5 (Table 4). These estimates are clearly limited by the retrospective nature of the survey and reflect physician’s opinion and not patient perception.

**Table 4:** Ranking of disease severity according to physicians

Disease	Mean	SD
Osteopenia	*4.61*	*0,70*
Osteogenesis imperfecta	*4.45*	*0,60*
McCune-Albright syndrome	*3.95*	*0,50*
Achondroplasia	*3.85*	*0,82*
XLH	*3.55*	*0,50*
Autosomal dominant hypophosphatemic rickets (*FGF23* mutation)	*3.50*	*0,71*
Vitamin D deficiency	*1.80*	*0,63*

1 = Low impact; 5 = High impact

## Discussion

XLH is a rare inherited disease, characterized by rickets and abnormal renal phosphate losses. Unrestricted expression of the phosphaturic hormone fibroblast growth factor 23 (FGF23) secondary to *PHEX* mutations cause most symptoms. Animal studies demonstrate that inactivation of PHEX increases FGF23 levels, which downregulates sodium-dependent phosphate transporters and 1 alpha hydroxylation of 25(OH)-vitamin D in renal proximal cells. The mechanisms by which *PHEX* mutations cause FGF23 production are still poorly understood. However, experimental and clinical data show that neutralizing antibodies against FGF23 prevent most XLH symptoms, thus demonstrating that this hormone is the principal culprit in this disease [10, 11].

Symptoms are mainly related to rickets in children and osteomalacia in adults, which cause different complications. XLH during childhood manifests mainly with bone deformities (bowed legs, genu varum or valgus, rachitic rosary, dolichocephaly), short stature, bone pain and dental abnormalities. The diagnosis is often delayed, which can significantly impact on long-term outcome [5, 6].

Conventional therapy includes supplementation with active vitamin D analogues and with oral phosphate salts. In most patients, symptoms improve, but the disease is not cured by conventional therapy [2]. This is most likely related to direct effects of high FGF23 levels on peripheral tissues, which are not inhibited by conventional treatment and may even be enhanced, since FGF23 levels increase in treated children [2]. Moreover, treatment is limited by side effects, in particular hyperparathyroidism and nephrocalcinosis that frequently develop when using high doses of therapy.

The present study was conducted to evaluate the current status in the diagnosis and treatment of XLH in Italy. Overall, results are in line with available data in the literature, although with some noticeable differences.

The prevalence of XLH is estimated between 1.2–3.0/60,000 [12, 13]. The expected number of prevalent pediatric patients with XLH in Italy is 187–470, considering an overall population of 9.374.322 persons aged 0–17 years. Our survey included 150 children diagnosed over a period of 20 years in 10 Italian centers with high experience in treating XLH. This number is lower than expected, indicating that the disease is either underdiagnosed or often treated in less specialized centers.

The diagnosis of XLH is based on clinical, radiological and biochemical grounds and can be confirmed by demonstrating a *PHEX* mutation. A positive X-linked family history usually confirms the diagnoses. When genetic analysis is not available and family history is absent, the disease is presumed, but cannot be demonstrated. In other cohorts, positive family history is observed in approximately 60% of cases [14, 15], as opposed to 33% in our survey. Most patients in which genetic testing was performed however, had a positive mutation, including patients with negative family history. It is unlikely that de novo mutations are more frequent in Italy. Most likely, parents of many patients were poorly symptomatic and/or reliable family history could not be traced back from medical records.

Our patients had overt symptoms with similar frequencies to those reported in the literature, with the exception of Chiari malformation and craniosynostosis. These are probably underdiagnosed because radiologic investigations, including brain MRI, are not routinely performed in most centers.

Conventional treatment usually improves bone pain and other XLH features but carries the risk of causing hyperparathyroidism and/or nephrocalcinosis. In the clinical practice, PTH levels, urinary calcium excretion and renal ultrasound are evaluated at regular interval to adapt treatment. Overall, nephrocalcinosis has been reported in the literature in 30–70% of patients [13, 15–17]. In the vast majority of cases, renal function is preserved, but very long-term data are not available yet. Most publications report a positive relationship between oral phosphate doses and nephrocalcinosis, whereas the association with vitamin D therapy is less strong [13, 17–19]. No increase in the supersaturation of either calcium oxalate or phosphate in urines was observed in one study including 20 patients with XLH [20]. Conceivably, the incidence observed in the Italian cohort is lower than previously reported (34%) because physicians have learned to balance treatment to avoid this complication. Hydrochlorothiazide has been shown to decrease calciuria in XLH [21] and potassium citrate may help preventing calcium precipitation. The prescription of these treatments was not included in the survey.

The incidence of hyperparathyroidism was more difficult to estimate because PTH levels are known to fluctuate overtime. Due to the retrospective nature of the survey only tertiary hyperthyroidism was evaluated and was diagnosed in 6% of patients.

Likewise, indication for orthopedic surgery in XLH are not clearly established and surgical attitudes have changed over the years [22–25]. If possible, osteotomies are performed preferentially after completion of growth [22, 26]. The only consistent finding in our survey was the increased use of epiphisiodesis in most recent years in all centers, while the overall number of surgeries did not change substantially overtime.

As expected, XLH has a negative impact on patient’s life. Most patients treated with conventional therapy are not cured and continue to experience complications related to their disease. The majority of children in this survey had normal daily activities despite their physical impairment. The impact in adults could not be estimated, due to the limited number of patients. However, data in the literature show that QoL in these patients can be severely affected [7, 27].

## Conclusion

This survey investigated the epidemiology, diagnosis and treatment of XLH in Italy. Despite its limitations, it highlights the severity of the disease and lack of homogeneity in treatment attitudes among specialists. Most importantly, results show a number of unmet needs and the limitations of conventional treatment that cannot cure the disease. In addition, these may cause adverse events, particularly when high dosages are used.

New therapies with anti-FGF23 antibodies are likely to modify the prognosis of XLH in the future [28–30].

## Abbreviations

FGF23: Fibroblast growth factor23
PTH: ParaThyroid hormone
QoL: Quality of life
SD: Standard deviation
XLH: X-Linked Hypophosphatemic

## Footnotes

1 Participating centers included: Bambino Gesù Children’s Hospital – Rome (F.E. and M.C.), Borgo Roma Hospital-University of Verona – Verona (F.A.), Istituto Auxologico Italiano IRCCS – Milan (M.L.B. and I.C.), IRCCS Cà Granda Foundation-Maggiore Policlinico Hospital – Milan (C.E.V.), Giannina Gaslini Institute – Genoa (N.D.I. and M.M.), S. Orsola Malpighi University Hospital – Bologna (A.C., A.B. and F.B.), Regina Margherita Children’s Hospital, University of Turin – Turin (L.D.S. and D.T.), University Hospital of Pisa – Pisa (G.B.), IRCCS San Raffaele Hospital-Vita-Salute San Raffaele University – Milan (G.W. and S.M.), Careggi University Hospital-University of Florence – Florence (M.L.B.).
